# Investigating neural markers of Alzheimer's disease in posttraumatic stress disorder using machine learning algorithms and magnetic resonance imaging

**DOI:** 10.3389/fneur.2024.1470727

**Published:** 2024-11-07

**Authors:** Gabriella Yakemow, Tiffany A. Kolesar, Natalie Wright, Iman Beheshti, Eun Hyung Choi, Lawrence Ryner, Sarah Chaulk, Ronak Patel, Ji Hyun Ko

**Affiliations:** ^1^Department of Human Anatomy and Cell Science, University of Manitoba, Winnipeg, MB, Canada; ^2^PrairieNeuro Brain Research Centre, Health Sciences Centre, Kleysen Institute for Advanced Medicine, Winnipeg, MB, Canada; ^3^Undergraduate Medical Education, Rady Faculty of Health Sciences, University of Manitoba, Winnipeg, MB, Canada; ^4^Department of Radiology, Rady Faculty of Health Sciences, University of Manitoba, Winnipeg, MB, Canada; ^5^Department of Clinical Health Psychology, University of Manitoba, Winnipeg, MB, Canada; ^6^Biomedical Engineering, Price Faculty of Engineering, University of Manitoba, Winnipeg, MB, Canada

**Keywords:** Alzheimer's disease, posttraumatic stress disorder (PTSD), MRI, arterial spin labeling (ASL), machine learning

## Abstract

**Introduction:**

Posttraumatic stress disorder (PTSD) is a mental health disorder caused by experiencing or witnessing traumatic events. Recent studies show that patients with PTSD have an increased risk of developing dementia, including Alzheimer's disease (AD), but there is currently no way to predict which patients will go on to develop AD. The objective of this study was to identify structural and functional neural changes in patients with PTSD that may contribute to the future development of AD.

**Methods:**

Neuroimaging (pseudo-continuous arterial spin labeling [pCASL] and structural magnetic resonance imaging [MRI]) and behavioral data for the current study (*n* = 67) were taken from our non-randomized open label clinical trial (ClinicalTrials.gov Identifier: NCT03229915) for treatment-seeking individuals with PTSD (*n* = 40) and age-matched healthy controls (HC; *n* = 27). Only the baseline measures were utilized for this study. Mean cerebral blood flow (CBF) and gray matter (GM) volume were compared between groups. Additionally, we utilized two previously established machine learning-based algorithms, one representing AD-like brain activity (Machine learning-based AD Designation [MAD]) and the other focused on AD-like brain structural changes (AD-like Brain Structure [ABS]). MAD scores were calculated from pCASL data and ABS scores were calculated from structural T_1_-MRI images. Correlations between neuroimaging data (regional CBF, GM volume, MAD scores, ABS scores) and PTSD symptom severity scores measured by the clinician-administered PTSD scale for DSM-5 (CAPS-5) were assessed.

**Results:**

Decreased CBF was observed in two brain regions (left caudate/striatum and left inferior parietal lobule/middle temporal lobe) in the PTSD group, compared to the HC group. Decreased GM volume was also observed in the PTSD group in the right temporal lobe (parahippocampal gyrus, middle temporal lobe), compared to the HC group. GM volume within the right temporal lobe cluster negatively correlated with CAPS-5 scores and MAD scores in the PTSD group.

**Conclusion:**

Results suggest that patients with PTSD with reduced GM volume in the right temporal regions (parahippocampal gyrus) experienced greater symptom severity and showed more AD-like brain activity. These results show potential for early identification of those who may be at an increased risk for future development of dementia.

## Background

Posttraumatic stress disorder (PTSD) is a mental health disorder caused by experiencing or witnessing traumatic events that include exposure to actual or threatened death, serious injury, or sexual violence (1, 2). Symptoms consist of intrusive memories, avoidant behaviors, increased anxiety, and flashbacks (1, 3). A recent study has shown that 63% of Canadians above the age of 18 will likely experience at least one traumatic event and that approximately 7% of Canadians will screen positive for PTSD during their lifetime (4). Prevalence rates appear similar across the United States (5, 6). This risk of PTSD increases in vulnerable populations including: women (7), military personnel (8), first responders (9) and health care workers (10). As the population grows, the impact of PTSD will continue to increase—understanding this disorder and its neurobiological impact will be increasingly important for treatment.

PTSD is categorized as a Trauma- and Stressor-Related Disorder and does not have a clear neurobiological cause; yet, emerging evidence suggests that PTSD predisposes individuals to developing dementia later in life (1, 11–13). In fact, it has been suggested that the increased risk of developing dementia following a PTSD diagnosis is 1.5 to 2-fold compared to people without PTSD (14, 15). The increased risk of dementia in PTSD spans a variety of diagnoses including: frontotemporal dementia, vascular dementia, and Alzheimer's disease (AD) (16). The present study focuses on the most prominent cause of dementia (AD), which accounts for approximately 70% of all dementia cases (17).

In contrast to PTSD, which can occur at any age but on average begins in young adulthood to middle-age, AD symptoms typically present around age 65 (18). The biggest risk factor for developing AD is age −47% of people tested above the age of 85 had probable diagnoses of AD (19). PTSD and AD share common symptoms including negative changes in mood and cognition, personality changes, memory difficulties and alterations in arousal or reactivity symptoms (20, 21). A recent study suggests that PTSD symptom severity is associated with accelerated cognitive decline, a leading symptom of AD (22).

Although the neurobiological underpinnings of PTSD have been difficult to isolate, a recent meta-analysis identified several brain regions that have been shown to have reduced gray matter (GM) volume, including the medial frontal gyrus, posterior cingulate gyrus, hippocampus, amygdala, prefrontal cortex (PFC), and insula (23). Furthermore, significant correlations have been observed between increased PTSD symptom severity and decreased GM volume within the temporal lobe, and most commonly in the hippocampus (24–26). Interestingly, AD also typically shows neurodegeneration in the medial temporal lobe, hippocampus and amygdala (27–31). In addition to these memory-related brain regions, AD can also be characterized by decreased activity (cerebral blood flow [CBF] or metabolism) in the frontal cortex and posterior cingulate cortex (32–36) which are also commonly found in PTSD (37–40). These overlapping neuroimaging findings may point to a mechanism for the increased risk of AD following a PTSD diagnosis.

Currently, no biomarkers are available to identify which patients with PTSD will progress to AD. This study explores different neuroimaging modalities to investigate possible neural biomarkers of dementia in a high-risk population. In this study, we characterize GM volume and CBF differences between PTSD and HC groups, then apply two machine learning algorithms across two neuroimaging modalities to estimate AD-like Brain Structure (ABS) scores (41) and AD-like activity pattern (Machine learning-based AD Designation [MAD]) scores (42) in PTSD. MAD scores indicate how similar a patient's brain activity is to AD patterns of brain activity—the higher the score, the greater the similarity. The MAD algorithm was originally trained using fluorodeoxyglucose (FDG) positron emission tomography (PET) data of 94 patients with AD and 111 age-matched HC from the AD Neuroimaging Initiative (ADNI; http://adni.loni.usc.edu/) database. MAD has been shown to be compatible with CBF images derived from perfusion SPECT (42) and pCASL data (43). Furthermore, MAD scores have been shown to increase over time only in patients with mild cognitive impairment (MCI) who later progress to dementia—MAD scores of patients with stable MCI remained stationary (44). Finally, MAD scores were also elevated in patients with epilepsy experiencing cognitive decline, compared to cognitively intact patients with epilepsy (45), suggesting its utility in identifying at-risk individuals with comorbidity.

## Materials and methods

### Participants

A total of 67 participants aged 18–65 were recruited for the open-label, non-randomized parallel clinical trial (ClinicalTrials.gov Identifier: NCT03229915)—only baseline data were used for the present study. Two groups were included in this study: PTSD (*n* = 40) and HC (*n* = 27). Patients in the PTSD group were treatment-seeking individuals who had experienced a criterion A traumatic event. The HC group consisted of trauma-naïve (TNC; *n* = 15) and trauma-exposed (TEC; *n* = 12) participants; due to low sample size TEC and TNC groups were pooled. TEC participants met CAPS-5 Criterion A (i.e., exposure to a traumatic event), while obtaining a CAPS-5 total severity score of ≤ 5. Demographic data are provided in Table 1.

**Table 1:** Demographic and CAPS-5 data for PTSD and HC groups.

	**PTSD**	**HC**	***t* or χ^2^**	**df**	***p***
*N*	40	27			
Age (years)	40.0 (±11.9)	35.4 (±14.0)	−1.426	65	0.159
Sex (M:F)	14:26	11:16	0.227	1	0.634
Handedness (R:L)	33:6^†^	24:3	0.247	1	0.619
Education (years)	14.0 (±2.6)	16.5 (±3.2)	3.556	65	0.001^*^
CAPS-5	33.4 (±9.4)	0.8 (±1.5)	17.771	65	<0.001^*^
MAD	−0.516	0.000	1.376	65	0.174
ABS	0.184	0.229	0.831	65	0.409

^*^Significant at p < 0.05.^†^Data missing for one participant. MAD scores are z-score normalized. CAPS-5, Clinician-administered PTSD scale for the DSM-5; MAD, machine learning-based Alzheimer's disease designation; ABS, Alzheimer's disease brain structure.

Exclusion criteria for all participants included substance dependence not in remission for at least 3 months, uncontrolled bipolar or psychiatric disorder, history of panic attacks, heart disease, respiratory distress, or neurological conditions including traumatic brain injury (TBI), as well as any MRI contraindications (e.g., metal implants or pregnancy). The CAPS-5 interview was administered to all participants to assess PTSD diagnosis and symptom severity (see Table 1). This study was approved by the Biomedical Research Ethics Board of the University of Manitoba and performed according to regulations. Participants provided written informed consent prior to participating in the study and received an honorarium for their time.

### MRI acquisition

MRI scans were acquired from all participants to investigate brain structure and function. Scans were acquired using a 3 Tesla, 12-channel Siemens MAGNETOM Verio MRI scanner (Erlangen, Germany) at the Kleysen Institute for Advanced Medicine in Winnipeg, Canada. Imaging acquired during each session included an anatomical T_1_-weighted scan (MPRAGE sequence; TR/TE/TI = 2,300/3.02/900 ms; flip angle = 9°; field of view (FOV) = 256 mm × 256 mm with 1.00 mm × 1.00 mm × 1.00 mm resolution; 240 slices), a resting state functional ${\text{T}}_{2}^{*}$- weighted scan (scan duration = 11 min; results forthcoming elsewhere), and a pseudo-continuous arterial spin labeling scan (pCASL; TR/TE = 4,000/12 ms; flip angle = 90°; FOV = 240 mm × 240 mm with 3.8 mm × 3.8 mm × 5 mm resolution; 90 volumes; 20 slices; slice thickness = 5; inter-slice gap = 1 mm; labeling time = 2 s; post label delay time = 1.2 s; bandwidth = 3 kHz/pixel). M0 images were also acquired (TR/TE = 8,000/12 ms) to calibrate the pCASL images.

### Structural MRI analysis

Structural T_1_-weighted images were preprocessed using the CAT12.8.2 toolbox (r2170; https://neuro-jena.github.io/cat/index.html) using SPM12 software (version 6909, www.fil.ion.ucl.ac.uk/spm/). First, a robust bias correction was applied to the MRI scans to reduce intensity variations. Then, the scans were segmented into gray matter (GM), white matter, and cerebrospinal fluid maps and spatially normalized into Montreal Neurological Institute (MNI) space (voxel size 1.5 mm × 1.5 mm × 1.5 mm) using the DARTEL algorithm. Modulation was applied to keep the volume information accurate. For VBM analysis, we smoothed the modulated GM images with an 8 mm^3^ full-width at half-maximum (FWHM) Gaussian kernel to improve the signal-to-noise ratio.

In order to detect any morphological differences in GM between individuals with PTSD and HC, we conducted a two-sample *t*-test on the smoothed GM images using SPM12 software. Age, sex, and total intracranial volume (TIV) were included as covariates during the analysis. An explicit mask, comprised of an average of all participant's GM masks, was used to limit search regions inside the GM of the brain. Results are considered significant at a cluster-forming threshold of *p* < 0.001 (uncorrected) and a cluster-level threshold of *p* < 0.05, corrected using family-wise error (FWE). Individual-level mean GM volumes were extracted from the significant clusters for further correlational analyses.

We estimated the previously described ABS scores (41) utilizing the region-based morphometry feature in CAT12 to obtain whole-brain GM volumes based on the neuromorphometrics atlas (www.neuromorphometrics.com; 136 regions in total), along with subregions of the hippocampus (18 regions) (46) and the cerebellum (26 regions) (47). Additionally, cortical thickness (CT) measurements were assessed using the Desikan-Killiany-Tourville (DKT) atlas, which defines 34 regions of interest in each cerebral hemisphere (48). In order to adjust for variations in brain size, the volumes of each subject were normalized by respective TIV. TIV was determined using the CAT12 toolbox (49). Utilizing the aforementioned brain structure-describing information, ABS ranks the most relevant features for classifying AD vs. HC based on a support vector machine (SVM) (41). The details of ABS model replication and validation are included in the Supplementary material.

### CBF analysis

For the CBF analysis, pCASL images were preprocessed using the default parameters of the ASLtbx (https://www.cfn.upenn.edu/zewang/ASLtbx.php) (50). First, data were realigned, then co-registered to the structural T_1_-weighted image, segmented and spatially normalized to standard MNI space (using nonlinear normalization by applying the deformations from the structural CAT12 analysis), and smoothed with an 8 mm^3^ FWHM Gaussian kernel, as described previously (51). Mean CBF images were produced and contrasted between the groups using SPM12. Age and sex were included as covariates. An explicit mask using the average whole-brain map of all participants was used to limit search regions within the brain. Results are considered significant at a cluster-forming threshold of *p* < 0.001 (uncorrected) and a cluster-level threshold of *p* < 0.05, corrected using FWE. Mean regional CBF values were extracted from significant clusters for further correlational analyses.

The smoothed mean CBF images were further assessed using our previously developed MAD algorithm (available at: https://www.kolabneuro.com/software1) (42). In the original work, out of the five different algorithms tested, the SVM with iterative single data algorithm (ISDA) was the best-performing machine (84% sensitivity and 95% specificity) for distinguishing between AD and HC—this algorithm was used in the present study (42).

### Statistical evaluation

Statistical analyses were conducted using the Statistical Package for the Social Sciences (SPSS; IBM Corp., version 27.0, Armonk NY) software. Demographic data were assessed using independent *t*-test or chi^2^, as appropriate (Table 1). Additionally, correlations between neuroimaging data (significant clusters in GM and CBF whole-brain analyses and MAD and ABS scores) and CAPS-5 total severity scores were assessed. Results were considered significant at *p* < 0.05.

## Results

PTSD and HC groups did not significantly differ for age or sex (Table 1). CAPS-5 scores and years of education significantly differed between groups (Table 1)—the PTSD group had higher PTSD symptom severity scores (*t*_(65)_ = 17.771, *p* < 0.001), as expected. Additionally, the PTSD group on average had fewer years of education (*t*_(65)_ = 3.556, *p* = 0.001). Sex, age, and TIV (GM volume analyses only) were used as covariates throughout the study.

### GM volumes

**Figure 1:**
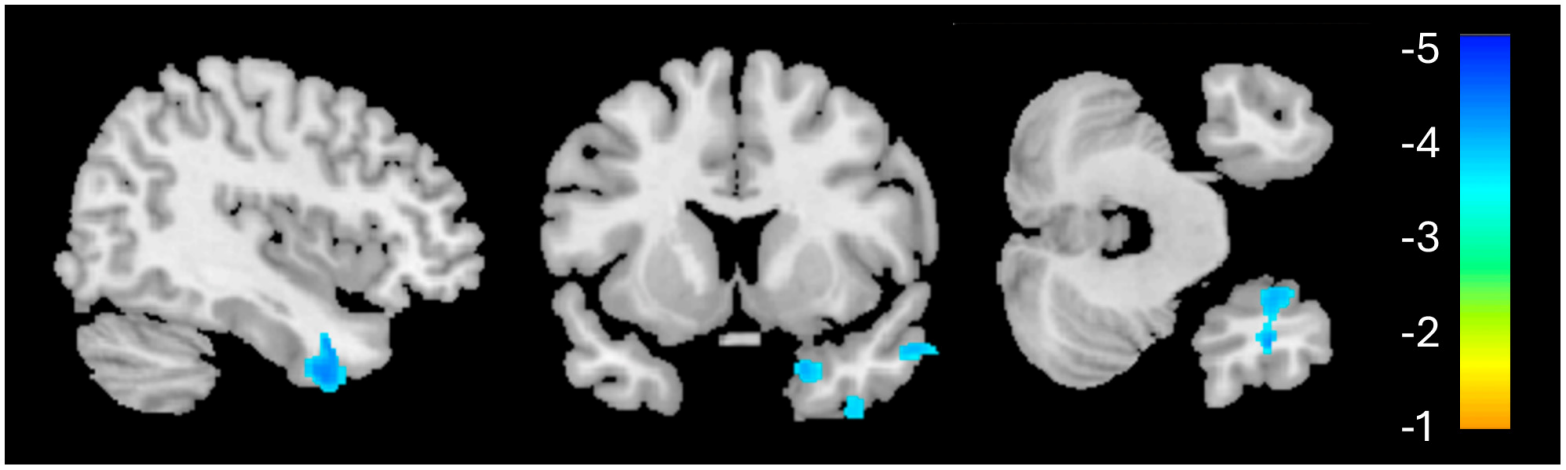
Whole-brain voxel-based morphometry results for the PTSD > HC contrast. The significant cluster (1,155 voxels) is identified in blue, indicating reduced volume in this region for the PTSD group, relative to HC.

**Figure 2:**
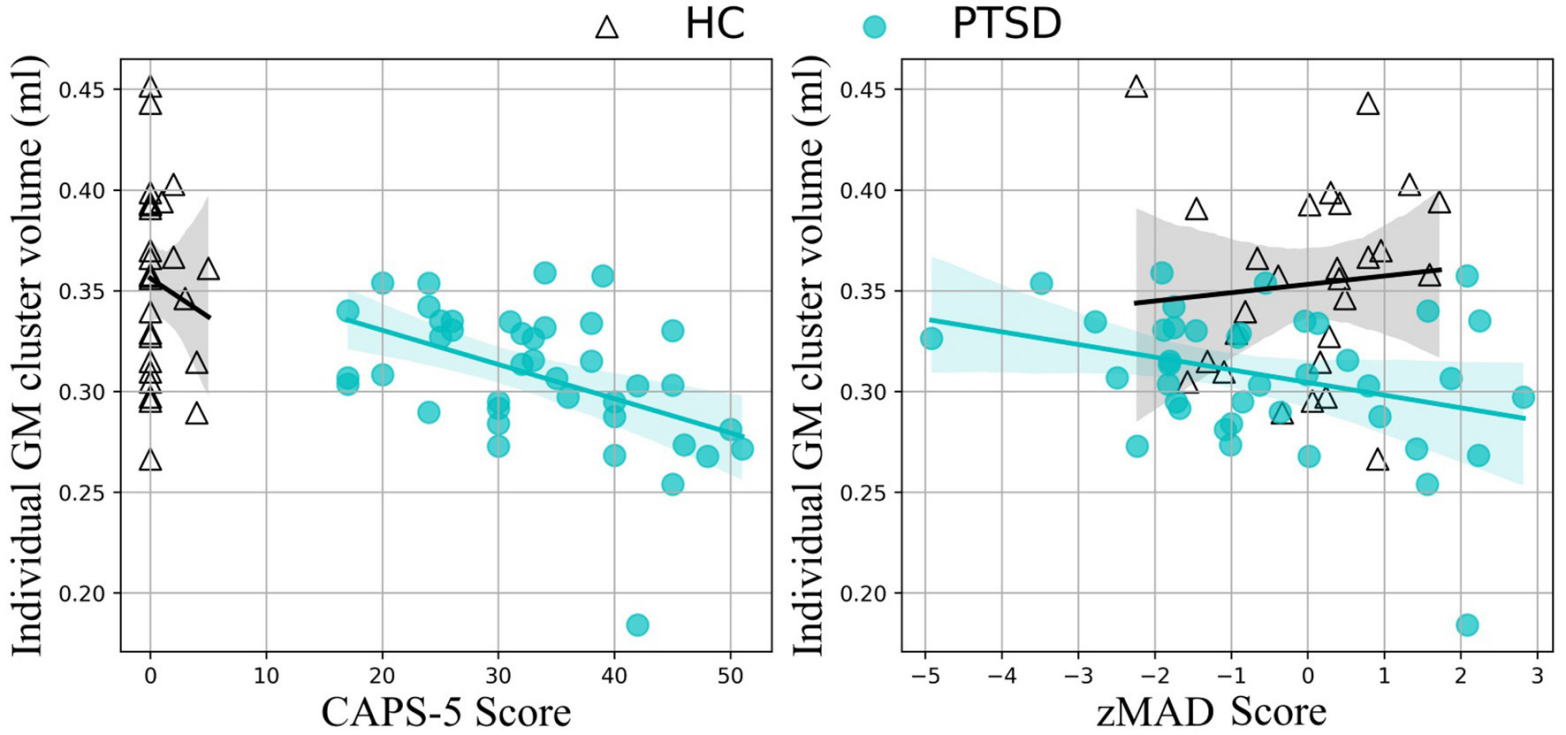
Correlation results. Medial temporal lobe volume correlates with CAPS-5 scores **(left)** and standardized MAD scores **(right)** in the PTSD group (teal, filled circles), but not the HC group (black, empty triangles).

Reduced volume was observed in the right middle temporal gyrus/parahippocampal gyrus for the PTSD group, compared to the HC group (Table 2, Figure 1). No regions showed significantly larger volume for the PTSD group, compared to the HC group. GM volume of the middle temporal gyrus cluster correlated with CAPS-5 total symptom severity scores (*r* = −0.461; *p* = 0.005; Figure 2).

**Table 2:** Whole-brain volume-based morphometry differences between groups (PTSD > HC), using age, sex, and total intracranial volume as covariates.

**Region**	**BA**	**Voxels**	***p*-value**	***t*-value**	**Peak coordinates**		
					**X**	**Y**	**Z**
Right middle temporal gyrus	20, 21	1,155	0.010^*^	−4.43	39	6	−33

^*^Significant at p < 0.05, FWE corrected for multiple comparisons. BA, Brodmann area.

ABS scores did not show any significant difference between the PTSD and HC groups (t_(65)_ = 0.831, *p* = 0.409). Further evaluation using a proportions z-test indicated that 11.1% (3/27) of the HC group and 7.5% (3/40) of the PTSD group were classified as AD-like, resulting in a *z*-statistic of 0.508, *p* = 0.612 (proportional difference = 3.61%, 95% CI = [−10.78%, 18.00%], Cohen's d = 0.1259), indicating no statistically significant difference between the groups.

### CBF

**Figure 3:**
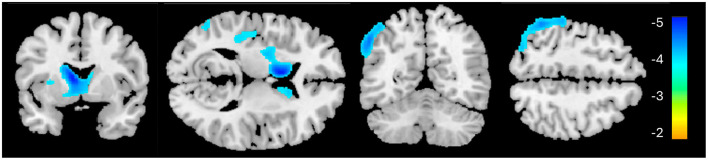
Whole-brain cerebral blood flow results for the PTSD > HC contrast. Significant clusters are identified in blue, indicating reduced cerebral blood flow in these regions for the PTSD group, relative to HC.

The PTSD group showed significantly lower CBF in two clusters, primarily in the left hemisphere, compared to the HC group (Table 3, Figure 3). The first cluster (3,933 voxels) had peak coordinates in the caudate and striatum and extended to the insula, parahippocampal gyrus, inferior frontal gyrus, limbic lobe and amygdala. The second cluster (4,069 voxels) peaked within the middle temporal gyrus and inferior parietal lobule and extends to the pre- and postcentral gyri. There was no significantly increased CBF for the PTSD group, compared to the HC group. The mean CBF values of either cluster did not correlate with CAPS-5 total severity scores.

**Table 3:** Whole-brain cerebral blood flow differences between groups (PTSD < HC), using age and sex as covariates.

**Region**	**BA**	**Voxels**	***p*-value**	***t*-value**	**Peak coordinates**		
					**X**	**Y**	**Z**
(1) Left Caudate/Striatum	13, 45, 47	3,933	0.004^*^	5.06	−8	9	10
(2) Left middle temporal gyrus/inferior parietal lobule	7, 39, 40	4,069	0.003^*^	4.45	−48	−75	28

^*^Significant at p < 0.05, FWE corrected for multiple comparisons. BA, Brodmann Area.

MAD scores did not significantly differ between groups [*t*_(65)_ = 1.376, *p* = 0.174]. At the individual level, five of the PTSD patients showed MAD scores (z-score) higher than 1.96 (*p* < 0.05), compared to none of the HC participants (χ^2^ = 3.647, *p* = 0.076). MAD scores correlated with GM volume in the significant middle temporal gyrus cluster identified above (*r* = −0.423; *p* = 0.010; Figure 2). A non-significant trending correlation was also observed between MAD scores and CAPS-5 total severity scores (*r* = 0.302, *p* = 0.065). Other correlational analysis results are summarized in Supplementary Table S1.

## Discussion

The goal of the present study was to find a novel non-invasive early neurological marker of AD in PTSD. We used machine learning algorithms (MAD and ABS) paired with neuroimaging and clinical variables to investigate these markers. The combination of these algorithms and PTSD symptom severity scores (CAPS-5) allowed us to identify a significant region of decreased brain volume in PTSD, associated with greater similarity to AD resting brain activity. Interestingly we noticed these markers in a PTSD participant group whose ages are younger than the typical age of diagnosis of AD.

As expected, and consistent with diagnostic criteria, there was a significant difference in CAPS-5 total symptom severity scores between the PTSD group and HC group, indicating increased PTSD symptom severity in the PTSD group (1, 52, 53). The CAPS-5 total severity scores were used as our primary psychiatric variable throughout the study. Additionally, education significantly differed between groups (Table 1), which is not surprising given that many of the participants in the PTSD group had careers as public safety personnel, typically requiring fewer years of formal education than our HC cohort, which had higher than average years of education.

Structural neuroimaging results identified one region of significantly reduced GM volume for the PTSD group, compared to the HCs (Figure 1). This middle temporal gyrus cluster included the parahippocampal cortex and Brodmann's areas (BA) 20 and 21 (Table 2). BA 20 is typically associated with processing visual information and memory association while BA 21 is involved in semantic memory processing, language processing and visual perception (54). Taken together, reduced volume within these regions may be associated with the experience of traumatic events and the ability to recall these memories (3, 55), and may be involved in symptoms such as flashbacks and accurate memory retention of traumatic events (1, 52). This result of decreased volume in the middle temporal lobe, more specifically the hippocampus and surrounding regions, is a common finding among PTSD structural neuroimaging results (56–58).

Interestingly, GM volume within this region was significantly, negatively correlated with CAPS-5 scores—smaller volume in this region was associated with greater symptom severity—a finding corroborated in the literature (24, 25). Furthermore, reduced GM volume in the temporal lobe cluster also correlated with MAD scores. In AD, the middle temporal lobe is the first region to begin showing neurodegeneration (30, 59), corresponding to the defining symptom of AD: memory deterioration (60). Although a causal role cannot be determined by the present study, it may be the case that individuals with reduced volume in this middle temporal region may be more susceptible to developing AD (as well as PTSD). Alternatively, trauma and the effects of PTSD may cause neurodegeneration in this region that increases the risk of dementia later in life. However, it is interesting to note that recently, psychological trauma in an animal model induced GM volume reduction in the hippocampus and globus pallidum (61). Further large-scale longitudinal investigations such as the UK Brain Bank (https://ukbbn.brainsfordementiaresearch.org) and Biofinder (https://biofinder.se/) should be used to shed more light on causality.

Topographically, MAD scores are determined by the overall whole-brain metabolic pattern, which is projected to a hyperplane used to differentiate between AD and HC (42). This hyperplane was largely characterized by hypometabolism in temporal regions. Therefore, it is not surprising that the regional GM reduction in the temporal regions observed in the present study correlated with increasing MAD scores in PTSD (Figure 2). While it remains to be seen whether there is any causal relationship between the location of the GM deterioration and AD progression, the lack of significant correlation within the HC group suggests that the medial temporal lobe may be an important region for predicting AD-risk in PTSD.

None of the ABS scores, which focus on AD-like structural patterns, correlated with any other neuroimaging results (GM, CBF, or MAD) or symptom severity (CAPS-5). Although the regional GM deficiency revealed by VBM analysis (Figure 1) spatially overlapped with features used in ABS classification, it should be noted that it does not constitute that they reflect the same pathology. The local GM difference between HC and PTSD groups was highly localized and it primarily reflects the PTSD-related changes (hence correlated with CAPS-5). On the contrary, ABS scores did not differ between groups. The relatively small regional atrophy in the right middle temporal lobe in our *cognitively healthy* PTSD sample may have not been sufficient to affect ABS scores, which capture the entire brain structural changes associated with AD. Nevertheless, the MAD score was correlated with regional GM changes. This observation is in line with the finding that AD-specific structural changes appear to occur after the functional (and/or vascular) changes (62).

Previously, alterations in CBF have been shown in PTSD, including increased activation in the amygdala (63) and decreased activity in the ventromedial prefrontal cortex and the inferior frontal gyrus (64). Our current results identified decreased CBF within the left caudate and striatum; this large cluster additionally encompassed the inferior frontal gyrus and insula—these findings confirm previous CBF findings of decreased activity in these cortical regions (64–67). Interestingly, research suggests that the insula and inferior frontal gyrus have become target stimulation regions for improving attentional decline in AD (68).

The second cluster, showing significantly reduced CBF in the left middle temporal gyrus and inferior parietal lobule in the PTSD group corresponds with the frontoparietal network (FPN; also known as the central executive network), a resting state network associated with attention and executive processing. Previous neuroimaging studies showed both increased (69–71) and decreased activity (65, 72–75) within this region in PTSD. Discrepancy in activity within this region in the literature may be due to several factors: first, whether resting metabolic/CBF or task-related activity are considered, as outlined, second, some studies have found reduced activity within the left IPL in the acute stage following traumatic exposure, when exposed to trauma-related (76) or fear-learning stimuli (77). In fact, left IPL activity in the acute phase was negatively correlated with symptom severity 3 months later (77).

The regional CBF did not correlate with MAD scores. Similar to the above interpretation for the lack of significant correlation between regional GM volumes and ABS scores, the reduced regional CBF, which reflect PTSD-related changes, may have not been sufficient to influence the MAD scores, which reflect the risks of dementia. It should also be noted that CBF images are much noisier than GM images, the regional change of which has shown to be correlated with MAD scores.

Limitations of this study include that its design was cross-sectional in nature; longitudinal study is required to confirm if patients with these identified neurological patterns go on to develop dementia, and more specifically, AD. We also acknowledge that there may be a selection bias for the HC groups as many of these participants were recruited by word of mouth and advertisements within the hospital and university settings, which may contribute to the higher years of education observed in this sample.

We used MAD scores for assessing AD-associated brain activity changes and ABS for assessing AD-associated brain structural changes. MAD was correlated with GM volume reduction in the medial temporal area which was correlated with PSTD symptom severity measured by CAPS-5. ABS was not correlated with any other measures. Taken together, as symptom severity increases, the brain structure and function become more AD-like, in the PTSD group—it is possible that these individuals who have higher MAD scores may be more susceptible to developing AD in the future. The data presented in our study are more poignant when the average age of our PTSD sample (i.e., 40.0 years) is considered—neurodegeneration in AD typically begins around 55 years of age (78). Early identification of individuals at an increased risk for developing dementia later in life is important for implementing disease-modifying preventative care.

In the current study, we used machine learning for early detection of markers associated with AD in PTSD. This study may provide information that will allow for a better understanding of brain structure and function in PTSD and how it may relate to the progression and possible increased risk of AD and dementia. To our knowledge, this is the first study that uses machine learning to assess the defining characteristics of AD within a PTSD population using neuroimaging.

## Data Availability

The raw data supporting the conclusions of this article will be made available by the authors, without undue reservation.

## References

[1] American Psychiatric Association. Diagnostic and Statistical Manual of Mental Disorders : DSM-5-TR. Washington, DC: American Psychiatric Association Publishing. (2022).

[2] Merians AN Spiller T Harpaz-Rotem I Krystal JH Pietrzak RH. Post-traumatic Stress Disorder. Med Clin North Am. (2023) 107:85–99. 10.1016/j.mcna.2022.04.003 36402502

[3] Brewin C. Posttraumatic stress disorder and the brain. J Neurol Neurosurg Psychiatry. (2013) 84:e1–e1. 10.1136/jnnp-2013-306103.3

[4] Government of Canada. Cycle 2: Symptoms of posttraumatic stress disorder (PTSD) during the COVID-19 pandemic. Public Health Agency of Canada (2021). Available at: https://www.canada.ca/en/public-health/services/publications/diseases-conditions/cycle-2-symptoms-posttraumatic-stress-disorder-covid-19-pandemic.html (accessed October 29, 2024).

[5] Schein J Houle C Urganus A Cloutier M Patterson-Lomba O Wang Y. Prevalence of post-traumatic stress disorder in the United States: a systematic literature review. Curr Med Res Opin. (2021) 37:2151–61. 10.1080/03007995.2021.1978417 34498953

[6] Raut SB Marathe PA Van Eijk L Eri R Ravindran M Benedek DM. Diverse therapeutic developments for post-traumatic stress disorder (PTSD) indicate common mechanisms of memory modulation. Pharmacol Therapeut. (2022) 239:108195. 10.1016/j.pharmthera.2022.108195 35489438

[7] Resnick HS Kilpatrick DG Dansky BS Saunders BE Best CL. Prevalence of civilian trauma and posttraumatic stress disorder in a representative national sample of women. J Consult Clin Psychol. (1993) 61:984–91. 10.1037/0022-006X.61.6.984 8113499

[8] Yehuda R Hoge CW Mcfarlane AC Vermetten E Lanius RA Nievergelt CM. Post-traumatic stress disorder. Nat Rev Dis Primers. (2015) 1:15057–15057. 10.1038/nrdp.2015.57 27189040

[9] Lewis-Schroeder NF Kieran K Murphy BL Wolff JD Robinson MA Kaufman ML. Conceptualization, assessment, and treatment of traumatic stress in first responders: a review of critical issues. Harv Rev Psychiatry. (2018) 26:216–27. 10.1097/HRP.0000000000000176 29975339 PMC6624844

[10] Carmassi C Foghi C Dell'oste V Cordone A Bertelloni CA Bui E. PTSD symptoms in healthcare workers facing the three coronavirus outbreaks: what can we expect after the COVID-19 pandemic. Psychiatry Res. (2020) 292:113312. 10.1016/j.psychres.2020.113312 32717711 PMC7370915

[11] Yaffe K Vittinghoff E Lindquist K Barnes DE Covinsky KE Neylan T. Post-traumatic stress disorder and risk of dementia among U.S. veterans. Alzheimer's Dementia. (2009) 5:P104. 10.1016/j.jalz.2009.05.326 PMC2933793 20530010

[12] Monfort E Trehel G. Post-traumatic stress disorder secondary to Alzheimer's disease: Emergence of an underlying pathology in the oldest old. Annales médico psychologiques. (2017) 175:776–80. 10.1016/j.amp.2017.03.020

[13] Desmarais P Weidman D Wassef A Bruneau M-A Friedland J Bajsarowicz P. The interplay between post-traumatic stress disorder and dementia: a systematic review. Am J Geriatric Psychiat. (2020) 28:48–60. 10.1016/j.jagp.2019.08.006 31488352

[14] Günak MM Billings J Carratu E Marchant NL Favarato G Orgeta V. Post-traumatic stress disorder as a risk factor for dementia: systematic review and meta-analysis. Br J Psychiatry. (2020) 217:600–8. 10.1192/bjp.2020.150 32933591

[15] Song H Sieurin J Wirdefeldt K Pedersen NL Almqvist C Larsson H. Association of stress-related disorders with subsequent neurodegenerative diseases. JAMA Neurol. (2020) 77:700–9. 10.1001/jamaneurol.2020.0117 32150226 PMC7063561

[16] Mohlenhoff BS O'donovan A Weiner MW Neylan TC. Dementia risk in posttraumatic stress disorder: the relevance of sleep-related abnormalities in brain structure, amyloid, and inflammation. Curr Psychiatry Rep. (2017) 19:1. 10.1007/s11920-017-0835-1 29035423 PMC5797832

[17] Moya-Alvarado G Gershoni-Emek N Perlson E Bronfman FC. Neurodegeneration Alzheimer's disease (AD). What Can Proteomics Tell Us About the Alzheimer's Brain? Mol Cellular Proteom. (2016) 15:409–25. 10.1074/mcp.R115.053330 26657538 PMC4739664

[18] Awada AA. Early late-onset Alzheimer's disease: What are the differences? J Neurosci Rural Pract. (2015) 6:455–6. 10.4103/0976-3147.154581 26167048 PMC4481819

[19] Evans DA Funkenstein HH Albert MS Scherr PA Cook NR Chown MJ. Prevalence of Alzheimer's disease in a community population of older persons: higher than previously reported. JAMA. (1989) 262:2551–6. 10.1001/jama.1989.03430180093036 2810583

[20] Lancaster C Teeters J Gros D Back S. Posttraumatic stress disorder: overview of evidence-based assessment and treatment. J Clin Med. (2016) 5:105. 10.3390/jcm5110105 27879650 PMC5126802

[21] Weller J Budson A. Current understanding of Alzheimer's disease diagnosis and treatment. F1000Research. (2018) 7:1161. 10.12688/f1000research.14506.1 30135715 PMC6073093

[22] Prieto S Nolan KE Moody JN Hayes SM Hayes JP. Posttraumatic stress symptom severity predicts cognitive decline beyond the effect of Alzheimer's disease biomarkers in Veterans. Transl Psychiatry. (2023) 13:102. 10.1038/s41398-023-02354-0 36990983 PMC10060413

[23] Siehl S Zohair R Guldner S Nees F. Gray matter differences in adults and children with posttraumatic stress disorder: a systematic review and meta-analysis of 113 studies and 11 meta-analyses. J Affect Disord. (2023) 333:489–516. 10.1016/j.jad.2023.04.028 37086802

[24] Gilbertson MW Shenton ME Ciszewski A Kasai K Lasko NB Orr SP. Smaller hippocampal volume predicts pathologic vulnerability to psychological trauma. Nat Neurosci. (2002) 5:1242–7. 10.1038/nn958 12379862 PMC2819093

[25] Villarreal G Hamilton DA Petropoulos H Driscoll I Rowland LM Griego JA. Reduced hippocampal volume and total white matter volume in posttraumatic stress disorder. Biol Psychiat. (2002) 52:119–25. 10.1016/S0006-3223(02)01359-8 12114003

[26] Harnett NG Goodman AM Knight DC. PTSD-related neuroimaging abnormalities in brain function, structure, and biochemistry. Exp Neurol. (2020) 330:113331–113331. 10.1016/j.expneurol.2020.113331 32343956

[27] Frisoni GB Fox NC Jack CR Scheltens P Thompson PM. The clinical use of structural MRI in Alzheimer disease. Nature Rev Neurol. (2010) 6:67–77. 10.1038/nrneurol.2009.215 20139996 PMC2938772

[28] Vemuri P Jack CR. Role of structural MRI in Alzheimer's disease. Alzheimer's Res Therapy. (2010) 2:23. 10.1186/alzrt47 20807454 PMC2949589

[29] Deture MA Dickson DW. The neuropathological diagnosis of Alzheimer's disease. Mol Neurodegener. (2019) 14. 10.1186/s13024-019-0333-5 31375134 PMC6679484

[30] De Flores R Das SR Xie L Wisse LEM Lyu X Shah P. Medial temporal lobe networks in Alzheimer's disease: structural and molecular vulnerabilities. J Neurosci. (2022) 42:2131–41. 10.1523/JNEUROSCI.0949-21.2021 35086906 PMC8916768

[31] Kim J Jeong M Stiles WR Choi HS. Neuroimaging modalities in Alzheimer's disease: diagnosis and clinical features. Int J Mol Sci. (2022) 23:6079. 10.3390/ijms23116079 35682758 PMC9181385

[32] Syed GMS Eagger S O'brien J Barrett JJ Levy R. Patterns of regional cerebral blood flow in Alzheimer's disease. Nucl Med Commun. (1992) 13:656–63. 10.1097/00006231-199209000-00004 1448238

[33] Alsop DC Detre JA Grossman M. Assessment of cerebral blood flow in Alzheimer's disease by spin-labeled magnetic resonance imaging. Ann Neurol. (2000) 47:93–100. 10632106

[34] Roher AE Debbins JP Malek-Ahmadi M Chen K Pipe JG Maze S. Cerebral blood flow in Alzheimer's disease. Vasc Health Risk Manag. (2012) 8:596–611. 10.2147/VHRM.S34874 23109807 PMC3481957

[35] Ortner M Drost R Heddderich D Goldhardt O Müller-Sarnowski F Diehl-Schmid J. Amyloid PET, FDG-PET or MRI? - the power of different imaging biomarkers to detect progression of early Alzheimer's disease. BMC Neurol. (2019) 19:9. 10.1186/s12883-019-1498-9 31672138 PMC6822351

[36] Thakur M Snekhalatha U. Multi-stage classification of Alzheimer's disease from 18F-FDG-PET images using deep learning techniques. Physi Eng Sci Med. (2022) 45:1301–15. 10.1007/s13246-022-01196-2 36357627

[37] Schuff N Zhang Y Zhan W Lenoci M Ching C Boreta L. Patterns of altered cortical perfusion and diminished subcortical integrity in posttraumatic stress disorder: An MRI study. Neuroimage. (2011) 54:S62–8. 10.1016/j.neuroimage.2010.05.024 20483375 PMC2945438

[38] Garip B Ozdemir B. 2697 – Reduced glucose metabolism in left lateral parietal cortex of a posttraumatic stress disorder patient: a case report. Eur Psychiat. (2013) 28:1–1. 10.1016/S0924-9338(13)77312-7

[39] Zandieh S Bernt R Knoll P Wenzel T Hittmair K Haller J. Analysis of the metabolic and structural brain changes in patients with torture-related post-traumatic stress disorder (TR-PTSD) using 18F-FDG PET and MRI. Medicine (Baltimore). (2016) 95:e3387–e3387. 10.1097/MD.0000000000003387 27082610 PMC4839854

[40] Soncin L-D Faure S Mcgonigal A Horowitz T Belquaid S Bartolomei F. Correlation between FDG-PET brain hypometabolism and PTSD symptoms in temporal lobe epilepsy. Epilepsia. (2022) 63(7):e74-e79. 10.1111/epi.17300 35569022 PMC9546285

[41] Beheshti I Demirel H Matsuda H. Classification of Alzheimer's disease and prediction of mild cognitive impairment-to-Alzheimer's conversion from structural magnetic resource imaging using feature ranking and a genetic algorithm. Comput Biol Med. (2017) 83:109–19. 10.1016/j.compbiomed.2017.02.011 28260614

[42] Katako A Shelton P Goertzen AL Levin D Bybel B Aljuaid M. Machine learning identified an Alzheimer's disease-related FDG-PET pattern which is also expressed in Lewy body dementia and Parkinson's disease dementia. Sci Rep. (2018) 8. 10.1038/s41598-018-31653-6 30185806 PMC6125295

[43] Wright N Alhindi A Millikin C Modirrousta M Udow S Borys A. Elevated caudate connectivity in cognitively normal Parkinson's disease patients. Sci Rep. (2020) 10:17978–17978. 10.1038/s41598-020-75008-6 33087833 PMC7578639

[44] Beheshti I Geddert N Perron J Gupta V Albensi BC Ko JH. Monitoring Alzheimer's disease progression in mild cognitive impairment stage using machine learning-based FDG-PET classification methods. J Alzheimer's Dis. (2022) 89:1493–502. 10.3233/JAD-220585 36057825 PMC9661333

[45] He M Kolesar TA Goertzen AL Ng MC Ko JH. Do epilepsy patients with cognitive impairment have alzheimer's disease-like brain metabolism? Biomedicines. (2023) 11:1108. 10.3390/biomedicines11041108 37189726 PMC10135603

[46] Winterburn JL Pruessner JC Chavez S Schira MM Lobaugh NJ Voineskos AN. A novel in vivo atlas of human hippocampal subfields using high-resolution 3 T magnetic resonance imaging. Neuroimage. (2013) 74:254–65. 10.1016/j.neuroimage.2013.02.003 23415948

[47] Park MTM Pipitone J Baer LH Winterburn JL Shah Y Chavez S. Derivation of high-resolution MRI atlases of the human cerebellum at 3 T and segmentation using multiple automatically generated templates. Neuroimage. (2014) 95:217–31. 10.1016/j.neuroimage.2014.03.037 24657354

[48] Desikan RS Ségonne F Fischl B Quinn BT Dickerson BC Blacker D. An automated labeling system for subdividing the human cerebral cortex on MRI scans into gyral based regions of interest. Neuroimage. (2006) 31:968–80. 10.1016/j.neuroimage.2006.01.021 16530430

[49] Gaser C Dahnke R Thompson PM Kurth F Luders E. CAT-a computational anatomy toolbox for the analysis of structural MRI data. BioRxiv [preprint] 2011.495736. (2022). 10.1101/2022.06.11.495736 39102518 PMC11299546

[50] Wang Z Aguirre GK Rao H Wang J Fernández-Seara MA Childress AR. Empirical optimization of ASL data analysis using an ASL data processing toolbox: ASLtbx. Magn Reson Imaging. (2008) 26:261–9. 10.1016/j.mri.2007.07.003 17826940 PMC2268990

[51] Aljuaid M Booth S Hobson DE Borys A Williams K Katako A. Blood flow and glucose metabolism dissociation in the putamen is predictive of levodopa induced dyskinesia in Parkinson's disease patients. Front Neurol. (2019) 10:1217–1217. 10.3389/fneur.2019.01217 31824400 PMC6881455

[52] Weathers FW Bovin MJ Lee DJ Sloan DM Schnurr PP Kaloupek DG. The Clinician-administered PTSD scale for DSM-5 (CAPS-5): development and initial psychometric evaluation in military veterans. Psychol Assess. (2018) 30:383–95. 10.1037/pas0000486 28493729 PMC5805662

[53] Bryan CJ Russell HA Bryan AO Rozek DC Leifker FR Rugo KF. Impact of treatment setting and format on symptom severity following cognitive processing therapy for posttraumatic stress disorder. Behav Ther. (2022) 53:673–85. 10.1016/j.beth.2022.01.014 35697430

[54] Berti A Garbarini F Neppi-Modona M. Disorders of higher cortical function. In: Neurobiology of Brain Disorders. London: Elsevier. (2023) p. 613–634.

[55] Kroes MCW Whalley MG Rugg MD Brewin CR. Association between flashbacks and structural brain abnormalities in posttraumatic stress disorder. Eur Psychiat. (2011) 26:525–31. 10.1016/j.eurpsy.2011.03.002 21592738

[56] Kitayama N Vaccarino V Kutner M Weiss P Bremner JD. Magnetic resonance imaging (MRI) measurement of hippocampal volume in posttraumatic stress disorder: a meta-analysis. J Affect Disord. (2005) 88:79–86. 10.1016/j.jad.2005.05.014 16033700

[57] Gianaros PJ Jennings JR Sheu LK Greer PJ Kuller LH Matthews KA. Prospective reports of chronic life stress predict decreased grey matter volume in the hippocampus. Neuroimage. (2007) 35:795–803. 10.1016/j.neuroimage.2006.10.045 17275340 PMC1868546

[58] Greenberg MS Tanev K Marin M-F Pitman RK. Stress PTSD, and dementia. Alzheimer's and dementia. (2014) 10:S155–65. 10.1016/j.jalz.2014.04.008 24924667

[59] Xiao S Yang Z Su T Gong J Huang L Wang Y. Functional structural brain abnormalities in posttraumatic stress disorder: A multimodal meta-analysis of neuroimaging studies. J Psychiatr Res. (2022) 155:153–62. 10.1016/j.jpsychires.2022.08.010 36029627

[60] Kirshner HS. Memory loss, Alzheimer's disease, and dementia: a practical guide for clinicians, 3rd ed. Cognit Behav Neurol. (2022) 35:298–9. 10.1097/WNN.0000000000000323

[61] Ruat J Heinz DE Binder FP Stark T Neuner R Hartmann A. Structural correlates of trauma-induced hyperarousal in mice. Prog Neuro-Psychopharmacol Biol Psychiat. (2021) 111:110404–110404. 10.1016/j.pnpbp.2021.110404 34303744

[62] Iturria-Medina Y Sotero RC Toussaint PJ Mateos-Pérez JM Evans AC Weiner MW. Early role of vascular dysregulation on late-onset Alzheimer's disease based on multifactorial data-driven analysis. Nat Commun. (2016) 7:11934. 10.1038/ncomms11934 27327500 PMC4919512

[63] Sartory G Cwik J Knuppertz H Schürholt B Lebens M Seitz RJ. In search of the trauma memory: a meta-analysis of functional neuroimaging studies of symptom provocation in posttraumatic stress disorder (PTSD). PLoS ONE. (2013) 8:e58150–e58150. 10.1371/journal.pone.0058150 23536785 PMC3607590

[64] Hayes JP Hayes SM Mikedis AM. Quantitative meta-analysis of neural activity in posttraumatic stress disorder. Biol Mood Anxiety Disord. (2012) 2:9–9. 10.1186/2045-5380-2-9 22738125 PMC3430553

[65] Shin LM Mcnally RJ Kosslyn SM Thompson WL Rauch SL Alpert NM. Regional cerebral blood flow during script-driven imagery in childhood sexual abuse-related PTSD: A PET investigation. Am J Psychiatry. (1999) 156:575–84. 10.1176/ajp.156.4.575 10200737

[66] Yin Y Li L Jin C Hu X Duan L Eyler LT. Abnormal baseline brain activity in posttraumatic stress disorder: a resting-state functional magnetic resonance imaging study. Neurosci Lett. (2011) 498:185–9. 10.1016/j.neulet.2011.02.069 21376785

[67] Zhe X Liu K Mu Y-F Qi S Xi Y-B Du P. Decreased regional cerebral perfusion at resting state in acute posttraumatic stress disorder resulting from a single, prolonged stress event. Acad Radiol. (2016) 23:1083–90. 10.1016/j.acra.2016.05.002 27283071

[68] Chou Y-H Ton That V Sundman M. A systematic review and meta-analysis of rTMS effects on cognitive enhancement in mild cognitive impairment and Alzheimer's disease. Neurobiol Aging. (2020) 86:1–10. 10.1016/j.neurobiolaging.2019.08.020 31783330 PMC6995441

[69] Shaw ME Strother SC Mcfarlane AC Morris P Anderson J Clark CR. Abnormal functional connectivity in posttraumatic stress disorder. NeuroImage (Orlando, Fla). (2002) 15:661–74. 10.1006/nimg.2001.1024 11848709

[70] Nardo D Högberg G Flumeri F Jacobsson H Larsson SA Hällström T. Self-rating scales assessing subjective well-being and distress correlate with rCBF in PTSD-sensitive regions. Psychol Med. (2011) 41:2549–61. 10.1017/S0033291711000912 21672299

[71] Im JJ Namgung E Choi Y Kim JY Rhie SJ Yoon S. Molecular neuroimaging in posttraumatic stress disorder. Exp Neurobiol. (2016) 25:277–95. 10.5607/en.2016.25.6.277 28035179 PMC5195814

[72] Clark C Mcfarlane AC Morris P Weber DL Sonkkilla C Shaw M. Cerebral function in posttraumatic stress disorder during verbal working memory updating: a positron emission tomography study. Biol Psychiatry. (2003) 53:474–81. 10.1016/S0006-3223(02)01505-6 12644352

[73] Shin LM Orr SP Carson MA Rauch SL Macklin ML Lasko NB. Regional cerebral blood flow in the amygdala and medial prefrontalcortex during traumatic imagery in male and female vietnam veterans with PTSD. Arch Gen Psychiatry. (2004) 61:168. 10.1001/archpsyc.61.2.168 14757593

[74] Weber DL Clark CR Mcfarlane AC Moores KA Morris P Egan GF. Abnormal frontal and parietal activity during working memory updating in post-traumatic stress disorder. Psychiatry Res. (2005) 140:27–44. 10.1016/j.pscychresns.2005.07.003 16202566

[75] Molina ME Isoardi R Prado MN Bentolila S. Basal cerebral glucose distribution in long-term post-traumatic stress disorder. World J Biol Psychiat. (2010) 11:493–501. 10.3109/15622970701472094 20218804

[76] Ke J Zhang L Qi R Li W Hou C Zhong Y. A longitudinal fMRI investigation in acute post-traumatic stress disorder (PTSD). Acta radiol. (2016) 57:1387–95. 10.1177/0284185115585848 25995310

[77] Harnett NG Ference EW Wood KH Wheelock MD Knight AJ Knight DC. Trauma exposure acutely alters neural function during Pavlovian fear conditioning. Cortex. (2018) 109:1–13. 10.1016/j.cortex.2018.08.015 30265859 PMC6261786

[78] Ramachandran AK Das S Joseph A Shenoy GG Alex AT Mudgal J. Neurodegenerative pathways in alzheimer's disease: a review. Curr Neuropharmacol. (2021) 19:679–92. 10.2174/1570159X18666200807130637 32851951 PMC8573750

