# Supplementary material for: Investigating neural markers of Alzheimer's disease in posttraumatic stress disorder using machine learning algorithms and magnetic resonance imaging
Source: Front Neurol. 2024 Nov 7;15:1470727. doi: 10.3389/fneur.2024.1470727 (PMC11578870; doi:10.3389/fneur.2024.1470727)
Supplement: Supplementary file 1 [file Data_Sheet_1.pdf]

Supplemental Information:

Alzheimer's Disease-like Brain Structure Framework

To replicate the previously established Alzheimer's Disease-like brain structure (ABS) framework, we downloaded baseline structural MRI data of 402 healthy control (HC) and 284 Alzheimer's disease (AD) participants from the ADNI dataset (https://adni.loni.usc.edu/). The HCs were confirmed to have no cognitive impairment or neurological disorder as per ADNI criteria. To prevent any bias towards the majority class and lower the false negative rate in the prediction model, we chose 284 healthy controls at random from a group of 402.

The preprocessing was applied in the same fashion as the region-based morphometry analysis in CAT12. In total, 252 structural brain features (i.e., CT measurements, adjusted whole-brain GM volumes, adjusted hippocampal subregions, and cerebellar subregions) were used as input for the AD-like structural pattern framework. In order to diminish the effects of age and sex on the predicted values, we regressed out the effects of age and sex from the structural brain feature values by referencing the healthy control (HC) group. The dataset was split at random into two cohorts: a training set, made up of 90% of the data (255 healthy controls (HCs) and 255 Alzheimer's disease (AD) patients), and a validation set, which consisted of 10% of the data including 29 HCs and 29 ADs. Furthermore, the validation set was used to ensure an unbiased estimate of the model's performance on unseen data. The harmonization of data from multiple datasets was achieved using the ComBat technique in MATLAB (Fortin et al., 2018; https://github.com/Jfortin1/ComBatHarmonization).

To build the prediction model, we employed our previously validated AD identification framework, which incorporated a binary genetic algorithm (GA) to identify the best subset of features, as outlined in (Beheshti et al., 2017). The Fisher criterion was integrated into the objective function of the genetic algorithm. GA works by refining a collection of potential feature groups through multiple generations, all based on the idea of natural selection. At first, random sets of chromosomes (i.e., features) are generated, each representing a potential solution. These sets are then evaluated using a fitness function to determine their effectiveness. The GA then uses operations such as combining features from two groups (crossover) and changing features within a group (mutation) to generate new chromosomes (i.e., features). Eventually, the process aims to find the best possible set of features that maximize the distinction between two groups. The

parameters of the GA were configured with 50 populations, 200 iterations, a crossover probability of 0.8, and a mutation probability of 0.3. The optimal settings were identified by conducting a 10-fold cross-validation on the training data to achieve the best performance. The classification method used was a standard support vector machine (SVM) algorithm in MATLAB r2020b, specifically using the 'fitcsvm' function with a linear kernel and automatically determined kernel scale. The final prediction model was created using the entire training set and then applied to the validation set and our test data. The prediction performance on the training and validation sets was assessed through accuracy, sensitivity, and specificity metrics.

The ABS score effectively differentiated between AD patients and HCs in the training dataset using the 10-fold cross-validation method, achieving an accuracy of 92%, sensitivity of 89%, and specificity of 95%. Results were consistent on the validation set with an accuracy of 88%, sensitivity of 83%, and specificity of 83%.

References

Beheshti, I., Demirel, H., Matsuda, H., & Initiative, A. s. D. N. (2017). Classification of Alzheimer's disease and prediction of mild cognitive impairment-to-Alzheimer's conversion from structural magnetic resource imaging using feature ranking and a genetic algorithm. *Comput Biol Med*, *83*, 109-119. https://doi.org/10.1016/j.compbiomed.2017.02.011

Fortin, J. P., Cullen, N., Sheline, Y. I., Taylor, W. D., Aselcioglu, I., Cook, P. A.,…Shinohara, R. T. (2018). Harmonization of cortical thickness measurements across scanners and sites. *Neuroimage*, *167*, 104-120. https://doi.org/10.1016/j.neuroimage.2017.11.024

Supplementary Table 1: Partial Pearson correlation results for CAPS-5 scores, GM volume, activity, MAD scores, and ABS scores. Age, sex, education and total intracranial volume (GM volume only) were used as covariates for the correlation analyses.

**Correlations**	***df***	***r*-value**	***p*-value**
**PTSD**			
CAPS-5 x MTG volume	34	-0.461	0.005*
CAPS-5 x CBF caudate	35	0.053	0.755
CAPS-5 x CBF IPL	35	-0.091	0.592
CAPS-5 x ABS	34	-0.172	0.314
CAPS-5 x MAD	35	0.298	0.074
MTG volume x CBF caudate	34	-0.091	0.598
MTG volume x CBF IPL	34	-0.281	0.097
MTG volume x ABS	34	-0.198	0.240
MTG volume x MAD	34	-0.423	0.010*
CBF caudate x ABS	34	0.042	0.808
CBF caudate x MAD	35	0.139	0.411
CBF IPL x ABS	34	0.113	0.513
CBF IPL x MAD	35	0.217	0.196
ABS x MAD	34	0.163	0.342
**HC**			
CAPS-5 x MTG volume	21	-0.211	0.333
CAPS-5 x CBF caudate	22	0.097	0.653
CAPS-5 x CBF IPL	22	0.064	0.768
CAPS-5 ABS	21	-0.045	0.840
CAPS-5 x MAD	22	0.184	0.389
MTG volume x CBF caudate	21	0.065	0.768
MTG volume x CBF IPL	21	0.-279	0.197
MTG volume x ABS	22	0.026	0.906
MTG volume x MAD	21	0.049	0.823
CBF caudate x ABS	21	-0.166	0.449
CBF caudate x MAD	22	0.035	0.870
CBF IPL x ABS	21	-0.112	0.610
CBF IPL x MAD	22	-0.162	0.451
ABS x MAD	21	-0.229	0.292

ABS: Alzheimer's disease-like brain structure; CAPS-5: Clinician-administered PTSD scale for DSM-5; CBF: cerebral blood flow; IPL: inferior parietal lobule; MAD: machine-learning based Alzheimer's disease Designation; MTG: middle temporal gyrus

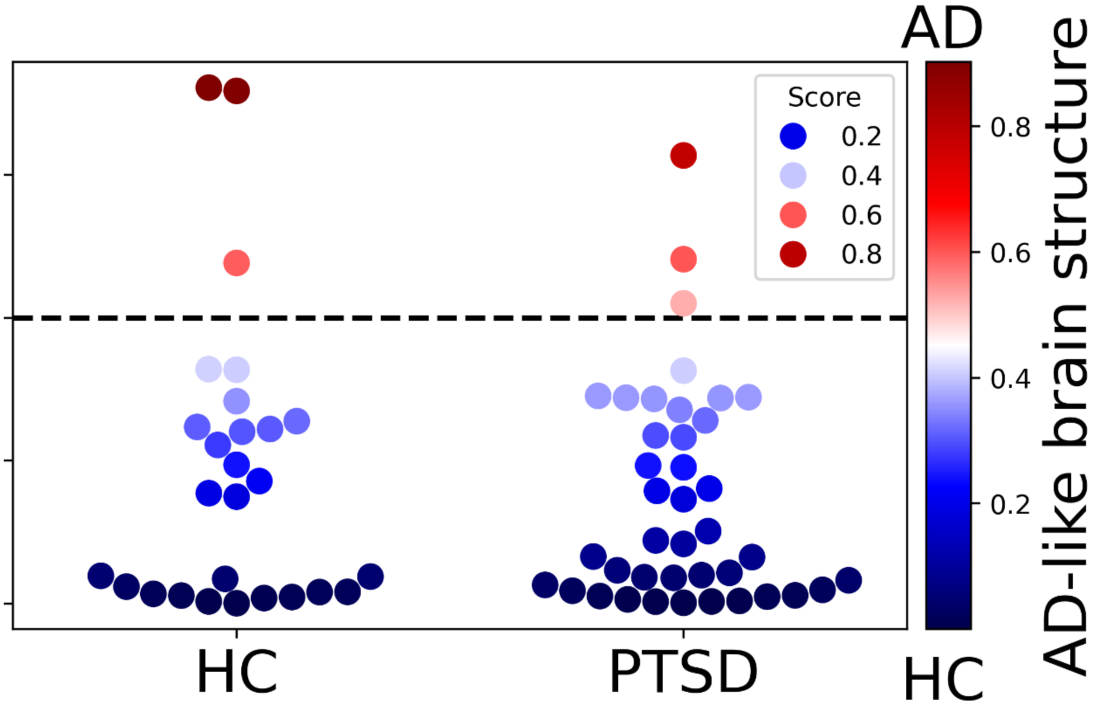

Supplementary Figure 1.

The scatter plot showing the Alzheimer's disease (AD)-like brain structure in test groups. The color bar provides a visual reference for the probability scores, ranging from 0 to 1. A horizontal dashed line at 0.5 denotes the midpoint for reference.
